# High COVID-19 Vaccine Hesitancy Among Healthcare Workers: Should Such a Trend Require Closer Attention by Policymakers?

**DOI:** 10.7759/cureus.17990

**Published:** 2021-09-15

**Authors:** Narmada Ashok, Kandamaran Krishnamurthy, Keerti Singh, Sayeeda Rahman, Md. Anwarul A Majumder

**Affiliations:** 1 Department of Pediatrics, Nalam Medical Centre and Hospital, Vellore, IND; 2 Department of Pediatrics, The University of the West Indies, Bridgetown, BRB; 3 Pediatric Intensive Care Unit, The Queen Elizabeth Hospital, The University of the West Indies, Bridgetown, BRB; 4 Faculty of Medical Sciences, The University of the West Indies, Bridgetown, BRB; 5 School of Medicine, American University of Integrative Sciences, Bridgetown, BRB

**Keywords:** india, tamil nadu, covid-19, healthcare workers, vaccine literacy, vaccine hesitancy, vaccine acceptance

## Abstract

Background

The newly developed coronavirus disease 2019 (COVID-19) vaccines are considered to be a powerful tool to contain the devastating pandemic. Healthcare workers (HCWs) are at the highest risk of exposure to COVID-19 and, therefore, they are the priority group for vaccination.

Objectives

The study aimed to examine the perceptions, attitudes, and acceptability of the COVID-19 vaccination among HCWs in India.

Study design

A cross-sectional pilot survey was conducted using an online questionnaire between 13 and 25 January 2021.

Results

Among 264 respondents, 40.2% of HCWs would receive the vaccine against COVID-19 if available and 32.2% were willing to take the vaccine after observing adverse effects in others. Infected members in the immediate social network (OR:2.15; 95%CI:0.426-10.844), COVID-19 knowledge (OR:5.113; 95%CI:0.974-26.853), the safety of vaccines (OR:7.608; 95%CI:2.618-22.11), and those who did not receive a flu vaccine last year (OR:2.612; 95%CI:1.120-6.091) were found to have a statistically significant association with vaccine acceptance. The main reasons to delay/refuse vaccination included: ‘quick vaccine development and compromised quality’ (43.7%) and ‘lack of trusted information regarding COVID-19’ (41.3%).

Conclusions

The finding showed a high rate of vaccine hesitancy among HCWs. Policymakers should take steps to increase public awareness and secure timely and affordable vaccines for the HCWs and general population with effective vaccine promotion campaigns.

## Introduction

It has been more than one and a half years since India reported its first case of coronavirus disease 2019 (COVID-19) on 27 January 2020, a Wuhan University Medical student who returned to Kerala. As of 13 August 2021, there were more than 32.1 million confirmed COVID-19 cases in India with 430,759 related deaths [1]. As there is no clinically proven treatment for COVID-19 infection, herd immunity can only be gained by high vaccination coverage worldwide [2-3]. Further vaccination remains the most effective way of controlling the disease, which unfortunately is challenged by delay or vaccine refusal by the general public [4].

India had fast-tracked the vaccine development process with the view to provide a lasting solution to the ongoing pandemic. With at least nine vaccines in different phases of development, Bharat Biotech and Serum Institute pioneered in their trials, and vaccines were made available for India, which were also to be rolled out in middle- and low-income countries (Global Alliance for Vaccines and Immunization (GAVI) countries) across the world [5].

When the vaccines became available, there was a hesitancy among the health care workers (HCWs) to take the vaccines in India [6-7]. Vaccine utilization and the attitude of HCWs play a key role in promoting compliance and adherence to vaccination schedules by vaccinees and their guardians [8]. Published evidence suggested hesitancy for vaccination among the general population was invariably linked to vaccine hesitancy amongst HCWs [4]. India had launched the world’s largest COVID-19 vaccination program starting with the frontline and health care workers on 16 January 2021. To date, more than 710 million people are vaccinated against COVID-19 in India [9].

With a sharp focus on the introduction and acceptance of vaccines during the current period of the pandemic, a pilot study was undertaken to examine the perceptions, attitudes, and acceptability of the vaccination among the health care workers in the state of Tamil Nadu (TN), India.

## Materials and methods

Study design and setting

Due to the limitations in doing face-to-face research because of the COVID-19 restrictions, we conducted an online descriptive cross-sectional study between 13 January and 25 January 2021 among HCWs who had access to the internet. The timing was chosen around the time of introduction of the vaccine in India and the sample population was HCWs working in TN, which is a southern state in India. Amongst all the states in India, TN has the largest HCWs and per capita health expenditures, and although not so affluent, it is known for its effective primary health care delivery models. Despite the effective health care services, TN continues to contribute a significant percentage of cases during the pandemic.

A convenient sampling technique was used. One of the researchers (MAAM) conducted a comprehensive literature review to identify relevant factors associated with COVID-19 vaccine acceptance and developed a questionnaire which was reviewed and finalized by the research teams from Bangladesh [10], Barbados, and India. The questionnaire included the following sections: (i) demographic information, (ii) knowledge of novel coronavirus (severe acute respiratory syndrome coronavirus 2 (SARS-CoV-2)) and COVID-19 [11], (iii) current flu/other vaccines behavior [11-12], (iv) acceptance of COVID-19 vaccination [11,13], (v) vaccine literacy knowledge [11,14], and (vi) COVID-19 vaccine perceptions and attitudes [11-12]. Questionnaires were circulated using Google Forms and the link was shared via WhatsApp and telegram instant messaging platforms.

The study was approved by the ethics committee of Nalam Medical Centre, Vellore, India (NMCHEC0007). Participation in the survey was entirely voluntary and informed consent was sought before participation.

Statistical analysis

The responses were extracted from Google Forms into an Excel sheet (Microsoft Corporation, Redmond, WA), the results were coded, and the data were analyzed using SPSS version 2020. Proportions and percentages were computed, and a chi-square test was used. Multinomial logistic regression was used to identify the factors contributing to the intention to vaccinate for COVID-19. The odds ratio and confidence interval were calculated and significant contributing factors were identified.

## Results

The questionnaires were circulated before the introduction of the vaccine and during the first phase of vaccination. The total number of respondents was 264, the majority were male (50.8%), the age group was 18-25 years (30.2%), and most were married (69.3%). Among the respondents, 80% were doctors, and among them, 58% were pediatricians and 21.6% were consultants from other specialties (Table 1).

**Table 1:** Demographic characteristics of HCWs (n=264) HCWs: health care workers

Variables		Frequency (%)
Gender	Male	136 (50.8%)
	Female	128 (48.5%)
Age (in years)	18-24	63 (23.9%)
	25-34	41 (15.5%)
	35-44	48 (18.2%)
	45 -54	66 (25%)
	55+	46 (17.4%)
Marital status	Married	183 (69.3%)
	Others (Single, divorced, or widowed)	81 (30.6%)
Occupation	Doctor (Other specialties)	57 (21.6%)
	Doctor (Pediatrician)	153 (58%)
	Nurse	15 (5.7%)
	Others	39 (14.7%)

**Figure 1:**
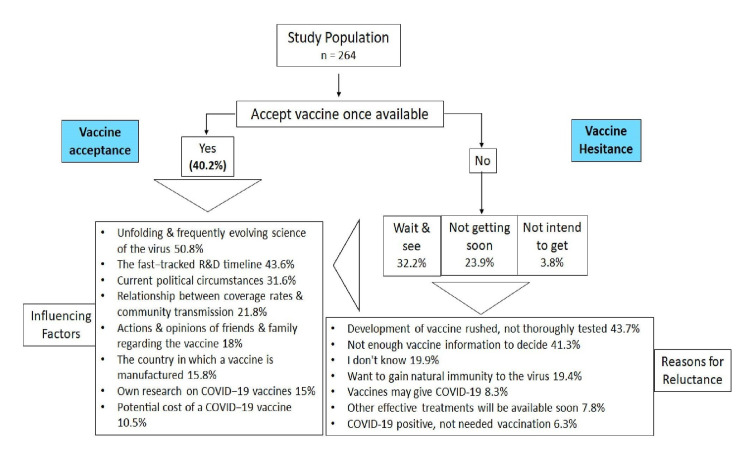
Acceptance/hesitancy of COVID-19 vaccine among HCWs HCWs: health care workers

Among the HCWs, 40.2% of them had the intention to get the vaccination immediately after it was available and 32.2% wanted to decide after seeing the effects of vaccination in others. Approximately one-quarter (23.9%) did not intend to get it anytime sooner and 3.8% did not intend to get the vaccine anytime (Figure 1). While analyzing the reasons for refusal or delaying the vaccination, it was observed that 43.7% felt that the development of the vaccine was rushed and 41.3% of HCWs felt they do not know everything about the vaccine to take it confidently (Figure 1). The frequently evolving science of COVID-19 vaccination was cited as the reason that influenced their opinion to get vaccinated by more than half of the respondents (Figure 1).

More than 90% of them felt they were knowledgeable about the COVID-19 infection. About 68.3% of respondents mentioned that they had someone in their immediate social network who was affected by COVID-19. With regards to the respondents’ behavior to the flu vaccine, 66.7% of the HCWs did not get vaccinated against influenza last year (2020), 41.3% of them wanted to be vaccinated against flu this year (2021), and 45.5% felt the flu vaccine was important every year. The majority of the respondents searched research articles (54.7%) or the internet (48.3%) for information regarding COVID-19 or about the vaccines.

To secure COVID-19 related information, the majority of the respondents (55.3%) consulted more than one source of information, although more than three-quarters (76.9%) of respondents ‘sometimes’ found the information they were looking for. More than half of the respondents (54.2%) ‘sometimes’ consulted with their colleagues and 45.5% relied ‘always’ on their colleagues for the COVID-related information. They considered the credibility of the sources only sometimes (53.8%) and felt that the information to help them get vaccinated was available only sometimes (57.6%). When asked questions pertaining to their perception of the safety and efficacy of COVID-19 vaccination, 52.3% of HCWs expressed their belief in the scientific vetting process of the vaccine development. However, only 23.1% felt vaccines were totally safe and 11.7% considered that vaccines were totally efficacious.

When asked about COVID-19 vaccine perceptions and attitudes, the majority of the respondents (73.5%) agreed that local authorities should check the efficacy of the vaccines prior to implementation and that COVID-19 vaccination should be made compulsory once available (46.6%). The majority were ready to pay for the vaccination (43.6%), recommend it to their patients, and felt children should also be vaccinated (45.85%).

The demographic variables and knowledge levels related to COVID-19 vaccines were analyzed for the intention to get vaccinated among HCWs - age (p-0.001), marital status (p-0.000), occupational level (p-0.000), and someone infected in the immediate social network (p-0.009) were significantly associated with their intention to get vaccinated (Table 2).

**Table 2:** Demographic variables and knowledge level of COVID-19 and intention for COVID-19 vaccination among HCWs HCWs: health care workers

Variables	Intend to take a vaccine					
	Do not intend on getting it soon but in the future. N (%)	Do not intend to ever get the vaccine. N (%)	Intend to get it as soon as possible. N (%)	Intend to wait to see how it affects others. N (%)	Total N (%)	p-value
Gender						
Female	30 (47.6%)	6 (60%)	49 (46.2%)	43(50.6%)	128 (48.5%)	0.821
Male	33 (52.4%)	4 (40%)	57 (53.8%)	42 (49.4%)	136 (51.5%)	
Age group						
>55yrs	9 (14.3%)	0 (0.0%)	30(28.3%)	7 (8.2%)	46 (17.4%)	0.001
18 -24	19 (30.2%)	4 (40%)	15 (14.2%)	25 (29.4%)	63 (23.9%)	
25-34	16 (25.4%)	3 (30%)	11 (10.4%)	11 (12.9%)	41 (15.5%)	
35-44	10 (15.9%)	2 (20%)	18 (17%)	18 (21.2%)	48 (18.2%)	
45 -54	9 (14.3%)	1 (10%)	32 (30.2%)	24 (28.2%)	66 (25%)	
Marital status						
Married	39 (61.9%)	3 (30%)	89 (84%)	52 (61.2%)	183 (69.3%)	0.000
Separated/divorced	0 (0%)	1 (10%)	1(10%)	0 (0%)	2 (0.8%)	
Single	23 (36.5%)	6 (60%)	14 (13.2%)	33 (38.8%)	76 (28.8%)	
Widowed	1 (1.6%)	0 (0.0%)	2 (1.9%)	0 (0.0%)	3 (1.1%)	
Occupation						
Doctors - Others	17 (27%)	2 (20%)	16 (15.1%)	22(25.9%)	57 (21.6%)	0.000
Doctor - Pediatrician	31 (49.2%)	2 (20%)	73 (68.9%)	47(55.3%)	153(58%)	
Nurse	1 (1.6%)	0 (0.0%)	10 (9.4%)	4 (4.7%)	15 (5.7%)	
Other	11 (17.5%)	6 (60%)	5 (4.7%)	12 (14.1%)	34 (12.9%)	
Pharmacist	2 (3.2%)	0 (0.0%)	0 (0.0%)	0 (0.0%)	2 (0.8%)	
Radiographer	1 (1.6%)	0 (0.0%)	2 (1.9%)	0 (0.0%)	3 (1.1%)	
Infected persons in the immediate social network						
Don’t know	10 (15.9%)	3 (30%)	1 (0.9%)	9 (10.6%)	23 (8.7%)	0.009
No	9 (14.3%)	3 (30%)	23 (21.7%)	16 (18.8%)	51 (19.3%)	
Yes but not yet confirmed	1 (1.6%)	0 (0.0%)	6 (5.7%)	3 (3.5%)	10 (3.8%)	
Yes confirmed	43 (68.3%)	4 (40%)	76 (71.7%)	57 (67.1%)	180 (68.2%)	
Knowledge level on corona vaccines						
Not knowledgeable	2 (3.2%)	1 (10%)	11 (10.4%)	3 (3.5%)	17 (6.4%)	0.155
Slight/moderate/extreme knowledgeable	61 (96.8%)	9 (90%)	95 (9.6%)	82 (96.5%)	247 (93.6%)	

The behavior of the HCW towards flu vaccination was also found to be significantly related to their intention to get the COVID-19 vaccine. The HCWs who had taken flu vaccines in 2020 and had an intention to take the flu vaccine this year were more likely to get vaccinated. Yearly flu vaccination was considered to be important by 77.7% of HCWs (Table 3). There was also a significant relationship with the intention to vaccinate others when the HCWs were confident in the scientific process (p-0.000); they felt that the vaccine was safe (p-0.000) and had good efficacy (p-0.000) (Table 4). This research also showed a significant relationship between the HCWs’ vaccination intention and the confidence in the scientific process (p-0.000), safety (p-0.000), and good efficacy of the vaccine (p-0.000).

**Table 3:** Behavior in relation to the flu vaccine and intention for COVID-19 vaccination among HCWs HCWs: health care workers

Variables	Intend to take the vaccine					
	Do not intend on getting it soon but in the future. N (%)	Do not intend to ever get the vaccine. N (%)	Intend to get it as soon as possible. N (%)	Intend to wait to see how it affects others. N (%)	Total N (%)	p-value
Vaccinated against the flu virus in 2020						
Not vaccinated	52 (82.5%)	8 (80%)	55 (51.9%)	61 (71.8%)	176 (66.7%)	0.000
Yes	11 (17.5%)	2 (20%)	51 (48.1%)	24 (28.2%)	88 (33.3%)	
Intention to get flu vaccination this year						
May be	27 (42.9%)	2 (20%)	28 (26.4%)	31 (36.5%)	88 (33.3%)	0.000
No	23 (36.5%)	7 (70%)	19 (17.9%)	18 (21.2%)	67 (25.4%)	
Yes	13 (20.6%)	1 (10%)	59 (55.7%)	36 (42.4%)	109 (41.3%)	
Importance to get flu vaccine every year						
Not important	14 (22.2%)	3 (30%)	14 (13.2%)	16 (18.8%)	47 (17.8%)	0.000
Somewhat important	36 (57.1%)	4 (40%)	29 (27.4%)	28 (32.9%)	97 (36.7%)	
Very important	13 (20.6%)	3 (30%)	63 (59.4%)	41 (48.2%)	120 (45.5%)	

**Table 4:** Perception of COVID-19 vaccination and intention to get vaccinated among HCWs HCWs: health care workers

Variables	Intention to take the vaccine					
	Do not intend on getting it soon, but in the future. N (%)	Do not intend to ever get the vaccine. N (%)	Intend to get it as soon as possible. N (%)	Intend to wait to see how it affects others. N (%)	Total N (%)	p-value
Confident in the scientific process						
Strongly disagree/disagree	10 (15.9%)	5 (50%)	5 (4.7%)	7 (8.2%)	27 (10.2%)	0.000
Neutral	41 (65.1%)	3 (30%)	21 (19.8%)	34 (40%)	99 (37.5%)	
Agree/strongly agree	12 (19%)	2 (20%)	80 (75.5%)	44 (51.8%)	138 (52.3%)	
Safety of the vaccine						
Not at all safe	2 (3.2%)	3 (30%)	1 (0.9%)	1 (1.2%)	7 (2.7%)	0.000
Little /moderate safe	2 5(39.7%)	1 (10%)	55 (51.9%)	55 (64.7%)	136 (51.5%)	
Completely safe	4 (6.3%)	2 (20%)	45 (42.5%)	10 (11.8%)	61 (23.1%)	
Don’t know	32 (50.8%)	4 (40%)	5 (4.7%)	19 (22.4%)	60 (22.7%)	
Effectiveness of the vaccine						
Not all effective	0 (0.0%)	4 (40%)	0 (0.0%)	3 (3.5%)	7 (2.7%)	0.000
Little/moderate	29 (46%)	1 (10%)	73 (68.9%)	59 (69.4%)	162 (61.4%)	
Totally effective	4 (6.3%)	1 (10%)	21 (19.8%)	5 (5.9%)	31 (11.7%)	
Don’t know	30 (47.6%)	4 (40%)	12 (11.3%)	18 (21.2%)	64 (24.2%)	

We further performed a multinomial regression to measure the association between COVID-19 vaccine intent and demographic factors of interest (Table 5). There was no significant association noted between willingness to vaccinate and age, gender, marital status, and perception of the effectiveness of the vaccine. Infected family members (OR:2.150; 95%CI:0.426-10.844), COVID-19 knowledge (OR:5.11; 95%CI:0.97-26.85), the safety of vaccines (OR:7.61; 95%CI:2.62-22.11), and last year’s flu vaccine non-recipients (OR:2.612; 95%CI:1.120-6.091) were found to have a statistically significant association with vaccine acceptance. The odds of delay of a vaccine was 0.7 times higher (OR:0.70; 95%CI:0.011-0.452) among respondents who didn’t know whether anybody in the immediate network was affected with COVID-19, and 27 times less likely to get vaccinated when they didn’t know about the safety of the vaccine (OR:27.727; 95%CI:0.92-840.49).

**Table 5:** Multinomial Logistic regression analysis for prediction of acceptance of COVID-19 vaccine among HCWs (n = 264) Reference category = not intending to vaccinate anytime sooner. *Values were statistically significant P<0.005. HCWs: health care workers

Variables	Characteristics	Intend to get as soon as possible	Intend to wait to see how it affects others	Do not intend to get vaccinated
Gender	Female	0.79 (0.35-1.74)	0.84 (0.38-1.88)	0.84 (0.17-4.25)
	Male (Ref)	-	-	-
Age	18-24 yrs	1.21 (0.34 -4.30)	2.85 (0.71-11.43)	2.55 (0.09-75.44)
	25 – 34 yrs	0.95 (0.13 -6.96)	0.72 (0.11-4.54)	0.42 (0.01 -16.61)
	45-54 yrs	0.69 (0.18-2.69)	1.03 (0.23 -4.72)	1.03 (0.04-27.72)
	>55%	1.16 (0.36-3.72)	2.96 (0.80-10.92)	1.84 (0.70-48.11)
	35-44 years (ref)	-	-	-
Anybody in the immediate network affected with COVID-19	Yes, but not confirmed	2.15 (0.43 -10.84)	1.1(0.29-4.22)	0.70 (.011 -0.45)*
	No	9.5 (0.64 – 140.56)	1.54 (0.11-21.31)	0.12 (0.00 -50.94)
	Don’t Know	4.65 (0.79 -27.25)*	1.95 (0.43 -8.86)	0.32 (0.05-2.22)
	Yes (ref)	-	-	-
Knowledge level of coronavirus	Extremely knowledgeable	5.11 (0.97-26.85)*	1.76 (0.47-6.63)	0.70(0.07-7.4)
	Somewhat/slightly knowledgeable	9.68 (0.99 – 95.05)*	1.78 (0.18-17.24)	1.19(0.02-60.98)
	Not knowledgeable	3.61 (0.62 -21.15)	1.92 (0.46-7.90)	0.64 (0.05-7.61)
	Moderately knowledgeable (Ref)	-	-	-
Vaccinated against flu virus in 2020	No	2.61 (1.12 -6.09)*	1.54 (0.63 -3.73)	1.87 (0.32-11.11)
	Yes (ref)	-	-	-
Safety of vaccines	Not safe	7.61 (2.62 -22.11)*	2.96 (1.13-7.8)***	1.85 (0.30 -11.44)
	Don’t know	0.95 (0.02-55.85)	0.38 (0.001-102.03)	27.73 (0.92-840.49)*
	Safe (ref)			

## Discussion

With India having a significant number of HCWs affected by COVID-19, and social media reflecting a lot of “conspiracy theories” among the general population [6-7], the attitudes and perception of HCWs are critical in vaccine promotion campaigns [8].

Vaccine hesitancy

The main findings of our study revealed that there was vaccine hesitancy among HCWs; less than half of the respondents stated that they would get vaccinated if a COVID-19 vaccine was available and one-third were likely to get vaccinated if no side-effects were observed after others were vaccinated.

Although a significant percentage of HCWs were knowledgeable (fair/moderate) with regard to COVID-19 and vaccination, still less than 40.2% were willing to get vaccinated as soon as it was available. Recent studies that were conducted worldwide on COVID-19 vaccine acceptance among the HCWs indicated that the range of acceptance was between 27.6% to 76.4% [4,6,8,10,13,15-20]. Our finding is consistent with a similar study conducted among HCWs in India before the introduction of the vaccine campaign, where an acceptance rate of 45% was observed [6]. Our finding is similar to that reported in a Bangladeshi study (43.8%) [10] but lower to the two USA studies (55% Los Angeles [13] and 57.5% New Mexico [19]) conducted among HCWs, which were conducted after a COVID-19 vaccine was developed and approved.

When comparing the results of vaccine acceptance in India, we found reports of a higher rate of acceptance of vaccination among the general population compared to HCWs in two studies that were conducted before any vaccine was developed - 86.3% and 79.5% [21-22]. In a large-scale study in the UK, 63.5% of the general population were willing to get vaccinated [23]. In contrast, a recent study was done in January 2021 during the vaccine roll-out phase in Bangladesh, one of the neighboring countries of India, reported vaccine acceptance rates of 25.5% [24]. A similar acceptance rate (37.4%) was found in a study conducted in Jordan, which was also conducted in November 2020 on the eve of mass vaccination [25]. Hence, it is possible that these studies, like our own, were done in the time frame when people had enough information regarding COVID-19 vaccines to take an informed decision reflecting the true acceptance rates. This indicates that a similar percentage of HCWs and the public have a hesitancy to get vaccinated immediately. While it can be acceptable for the public, it becomes necessary to analyze the hesitancy among HCWs. Probably, this contributory factor is now reflecting in the extent of vaccination in India, which is 38 doses for every 100 individuals and is lower than the world average of 60 doses [9].

Association with demographic variables

Among the demographic variables, age, marital status, and occupational level of the respondents had a significant association with the intention to get the vaccination. Age has been noted as a factor influencing vaccination in a global study on the potential acceptance of COVID-19 vaccination and in a study done in the USA. The distribution of age groups in the global multicentric study was similar to ours with the predominant age group being 18-54 years [26-27]. However, a study done in Australia did not find age to be a significant factor in influencing vaccination [28].

Association with a marital status similar to our study was found in the study conducted in Saudi Arabia in determinants of vaccine acceptance [29]. Even among the HCWs, the occupational level equating to a higher level of education and income, with respondents being predominantly doctors, was found to be associated with the intention to vaccinate in our study. A similar finding of significant association with higher educational status and income has been found in the recently published studies conducted in Australia [28] and Saudi Arabia [29].

Determinants of vaccine hesitancy

When the factors for the hesitancy were also analyzed, HCWs in our study felt that the science of COVID-19 vaccines was changing, and vaccine development was rushed. Interestingly, HCWs in Los Angeles also cited concerns regarding the fast-tracked nature of vaccine development and lack of transparency, which were the reasons for their hesitancy [13]. This was slightly different from the Jordanian population who believed that the pandemic itself was a conspiracy and did not trust any information [25]. Hence, it is imperative to address these issues with regards to the general mistrust about vaccination due to either the process or the intention of the government and manufacturers [13,15].

Multinomial regression analysis showed that the knowledge level of vaccination greatly influenced the decision to get vaccinated immediately. People who were ‘slightly knowledgeable’ were almost 10 times likely to be vaccinated, whereas, when they were not aware of the safety of the vaccine, they were 27 times unlikely to get vaccinated. This finding is similar to a large multicentric European study, where opinion about the safety of vaccines developed as the important independent factor for the determination of hesitancy of vaccination [27]. Barry et al. (2020) reported that vaccine safety was significantly associated with reporting willingness to be vaccinated among HCWs [20]. Fu et al. (2020) also found that vaccine safety was significantly associated with an increased probability of choosing vaccination in the general population [8].

The analysis also showed that last year’s flu vaccine non-recipients had a statistically significant association with vaccine acceptance and safety. However, a recent study conducted in France reported that previous influenza vaccination behavior was a predictor of COVID-19 vaccine acceptance [15]. The contrasting association between COVID-19 and flu vaccines among HCWs in our study may be in part due to the self-perceived risk of HCWs not getting infected by seasonal flu as described in a recent study done in India among HCWs, wherein it was observed that they perceived themselves as at low risk of contracting influenza, and their vaccination uptake was only 11% [30]. The flu vaccine coverage is also low in our study (33.3%). Decreased flu vaccine adherence may be associated with increased absenteeism due to illness, which may seriously impair normal clinical and COVID-19 services in indoor hospital care during this pandemic time.

Limitations

Our study has several limitations. The major limitations are the small sample size, a smaller number of respondents aged >55 years (17.4%), and unequal distribution of respondents throughout India, which restrict the generalizability of our results. Besides, the representativeness of the sample in terms of occupation as most of the respondents were physicians especially pediatricians. Though the study was sent across several groups, the response received was less in number, which is a common occurrence in the online survey. The sample size also contributed to our inability to get significant predictors for vaccination in multinomial regression. Hence, a larger study done in India involving multiple centers can help us predict the behavior of HCWs with regard to vaccination, which will reflect on the population. This is the need of the hour, as most countries are facing a resurgence.

## Conclusions

The results of this study show high rates of vaccine hesitancy among HCWs in India. As ultimate health care providers, their attitude is likely to influence public awareness at large. Currently, securing timely and equitable access to an affordable vaccine at the earliest opportunity is of utmost importance for the stakeholders of healthcare providers. Hence, a targeted approach toward raising awareness about the scientific process and transparency in vaccine development and manufacturing and the importance of mass vaccination may reduce vaccine hesitancy among HCWs and the general population.
